# Supplementary material for: MesoNet allows automated scaling and segmentation of mouse mesoscale cortical maps using machine learning
Source: Nat Commun. 2021 Oct 13;12:5992. doi: 10.1038/s41467-021-26255-2 (PMC8514445; doi:10.1038/s41467-021-26255-2)
Supplement: Supplementary file 1 — Supplementary Information [file 41467_2021_26255_MOESM1_ESM.pdf]

Supplementary Information:

Title:

MesoNet allows automated scaling and segmentation of mouse mesoscale cortical maps using machine learning.

Dongsheng Xiao, Brandon J. Forys, Matthieu P. Vanni, Timothy H. Murphy*

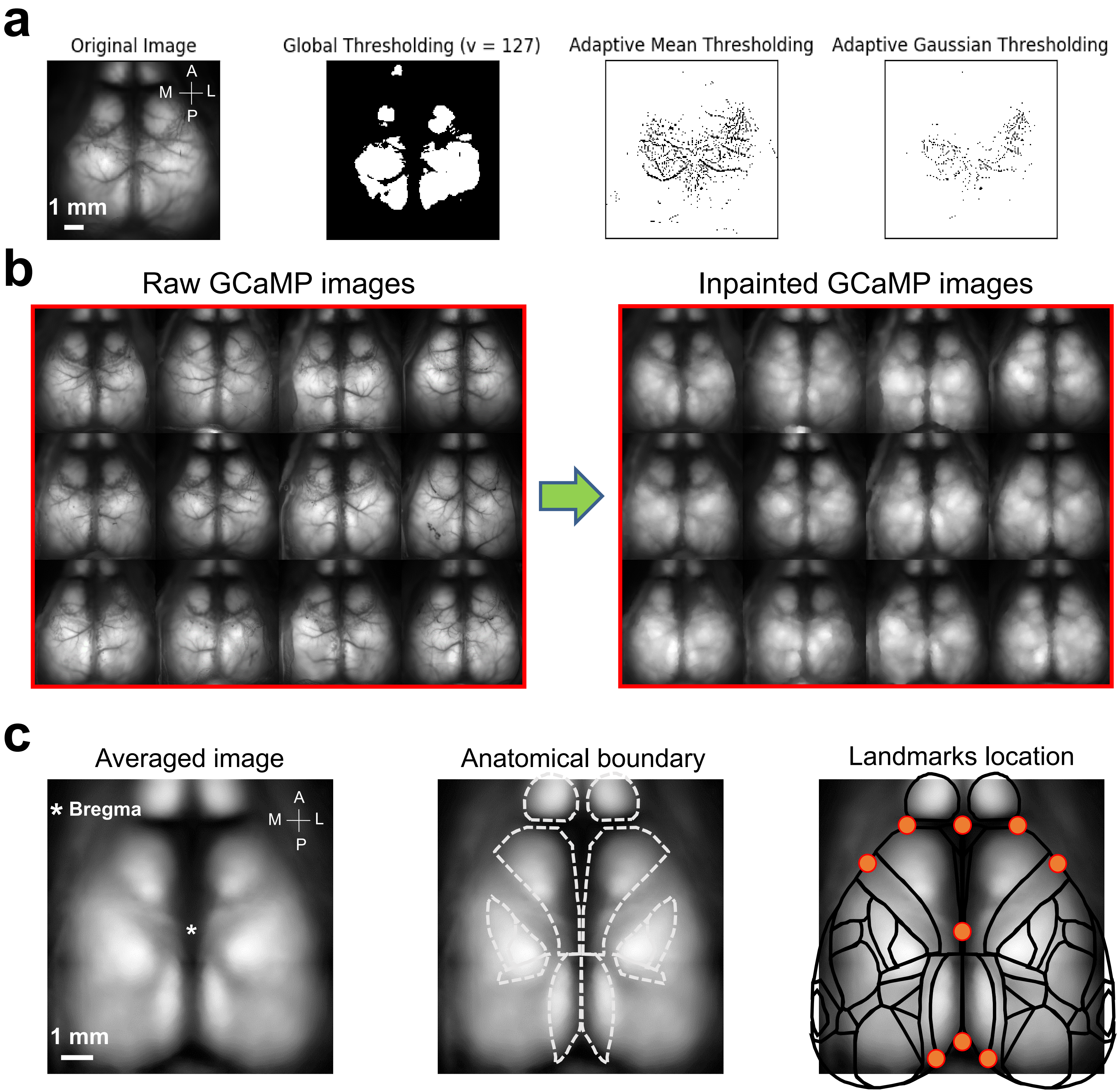
a
Original Image
A
M
L
P
1 mm
Global Thresholding (v = 127)
Adaptive Mean Thresholding
Adaptive Gaussian Thresholding
b
Raw GCaMP images
Inpainted GCaMP images
c
Averaged image
* Bregma
A
M
L
P
*
1 mm
Anatomical boundary
Landmarks location

Supplementary Fig. 1. Pre-processing of raw GCaMP images by inpainting reveals subtle structural features. a Results from global, adaptive mean (the threshold value is the mean of the neighborhood area minus the constant), and adaptive gaussian (the threshold value is a gaussian-weighted sum of the neighbourhood values minus the constant) thresholding show exclusion of undesired regions such as the outline of cortex and surface vasculature. **b** Examples of raw and post-processed GCaMP images by inpainting. **c** Averaged image of inpainted GCaMP images (*n* = 12 mice) shows distinct fluorescent regions corresponding to regions within the reference atlas. This helps to determine the common coordinates of landmarks on reference atlas and brain images.

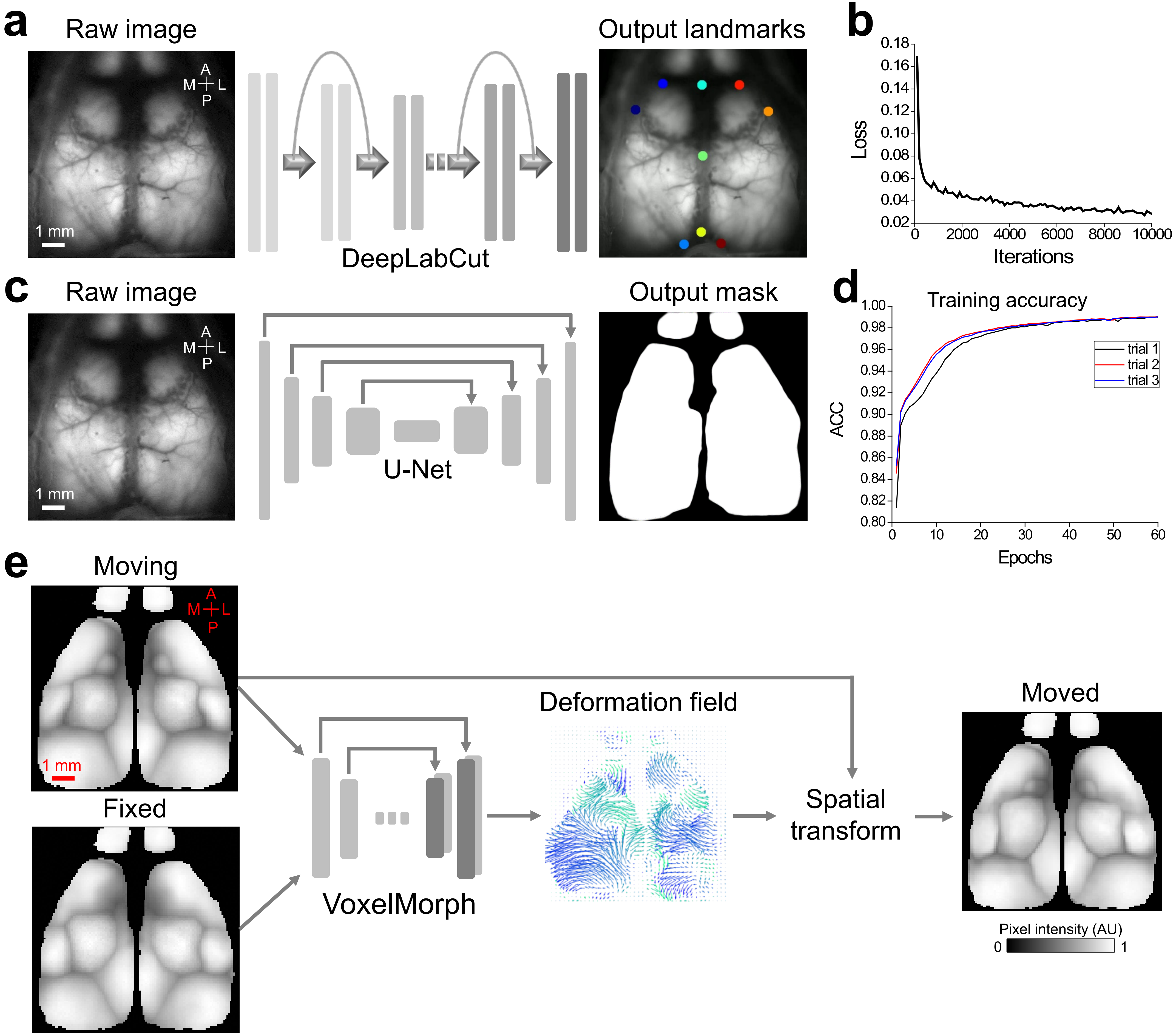
a
Raw image
A
M
L
P
1 mm
DeepLabCut
Output landmarks
b
Loss
Iterations
c
Raw image
1 mm
U-Net
Output mask
d
Training accuracy
ACC
Epochs
trial 1
trial 2
trial 3
e
Moving
1 mm
Fixed
VoxelMorph
Deformation field
Spatial transform
Moved
Pixel intensity (AU)
0
1

Supplementary Fig. 2. Summary of machine learning models used in MesoNet. a The architecture of deep neural network that is trained and used to predict the cortical landmarks on cortical raw fluorescent images. **b** The loss of our model converged near zero after approximately 10,000 iterations during training. **c** U-Net model is trained and used to delimit the cortical boundary on cortical raw fluorescent images automatically. **d** The training accuracy of our model reached 0.99 after 60 epochs of training (n = 3 trials). **e** Paired motif-based functional maps (MBFMs) were used to train VoxelMorph Model (unsupervised). The VoxelMorph model predicts a deformation field between a fixed MBFM image and a moving MBFM image. The spatial transformation can be used to deform a moving image to fit the fixed image.

a

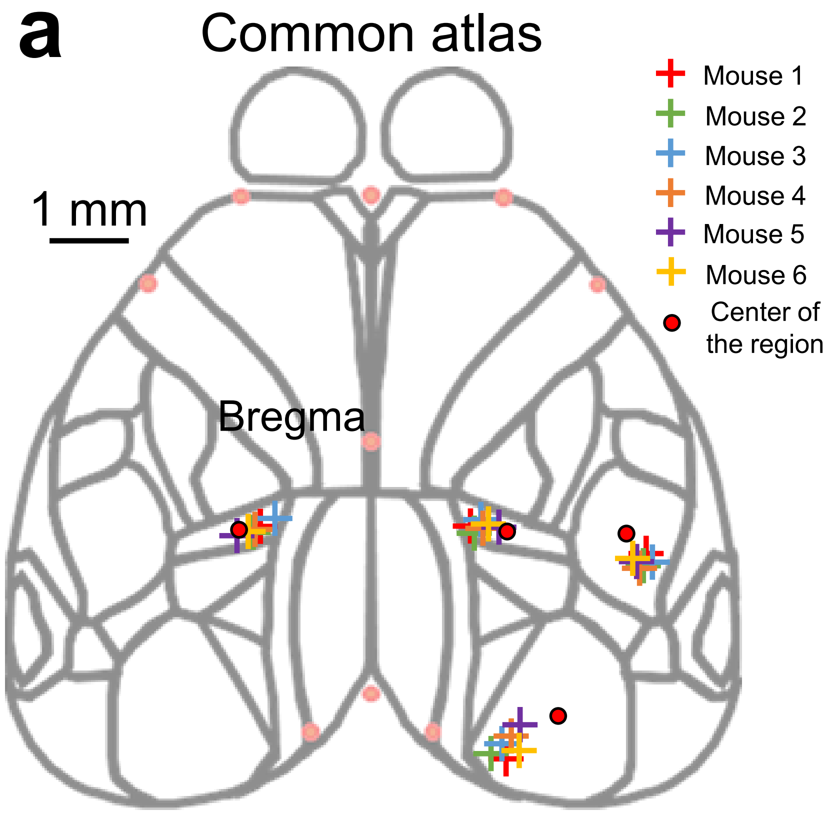

b

Sensory maps	Averaged coordinate (mm)
Left hemisphere tail region.	(-1.59, -1.21)
Right hemisphere tail region.	(1.60, -1.17)
Right hemisphere barrel cortex (upper whiskers).	(3.84, -1.63)
Right hemisphere visual cortex.	(2.08, -4.04)
Bregma.	(0, 0)

Supplementary Fig. 3. a Sensory map peaks on the common atlas ($n = 6$ mice). **b** Averaged coordinates of sensory map peaks.

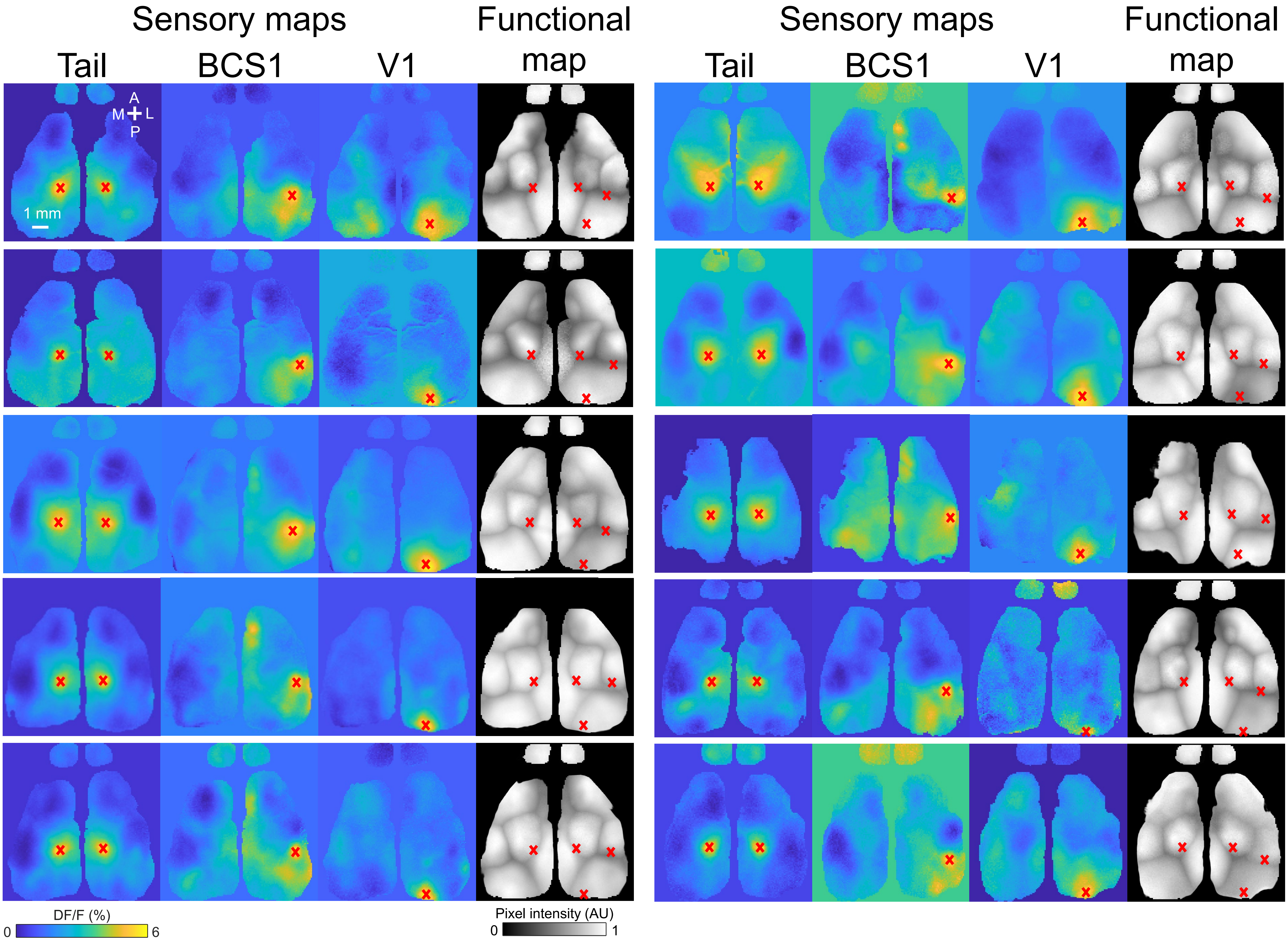

Sensory maps
Functional map
Tail
BCS1
V1
A
M
L
P
1 mm
Sensory maps
Functional map
Tail
BCS1
V1
DF/F (%)
0
6
Pixel intensity (AU)
0
1

Supplementary Fig. 4. Example sensory maps and MBFMs. Every panel shows sensory maps (Tail, V1, and BCS1) and MBFMs in the same mouse. The red cross is the peak activation of sensory maps. It shows a relatively stable location on MBFMs across mice (n = 10 mice).

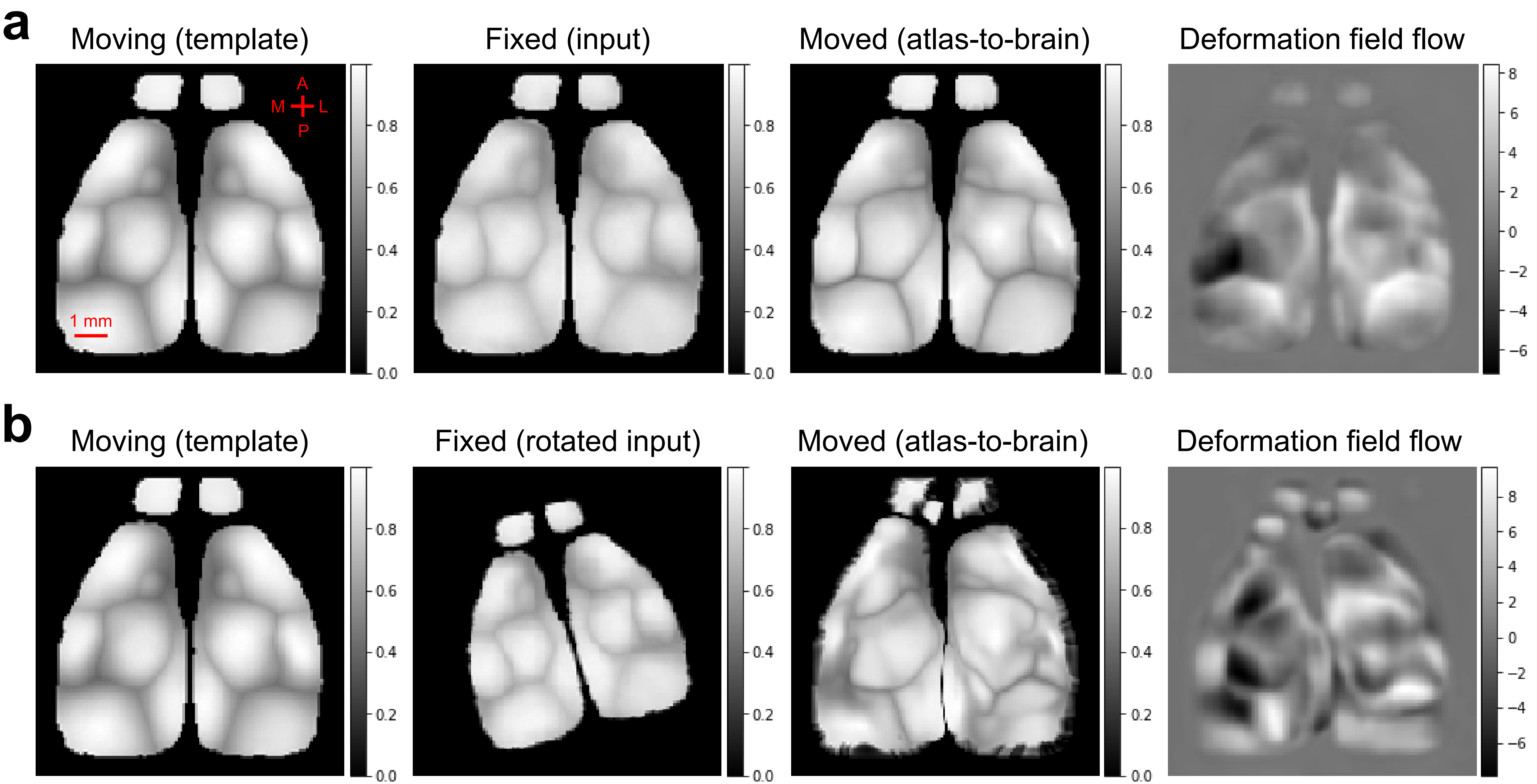

Supplementary Fig. 5. Example image deformation using VoxelMorph. a The fixed image (input MBFM in our pipeline, Fig. 7**c**) does not change. The template MBFM moves to it, and the template MBFM is aligned with the reference atlas (see Fig. 7**d**), so we can apply a deformation field flow to move the reference atlas to input MBFM (atlas-to-brain). **b** When the input MBFM is rotated, the performance of VoxelMorph becomes worse.

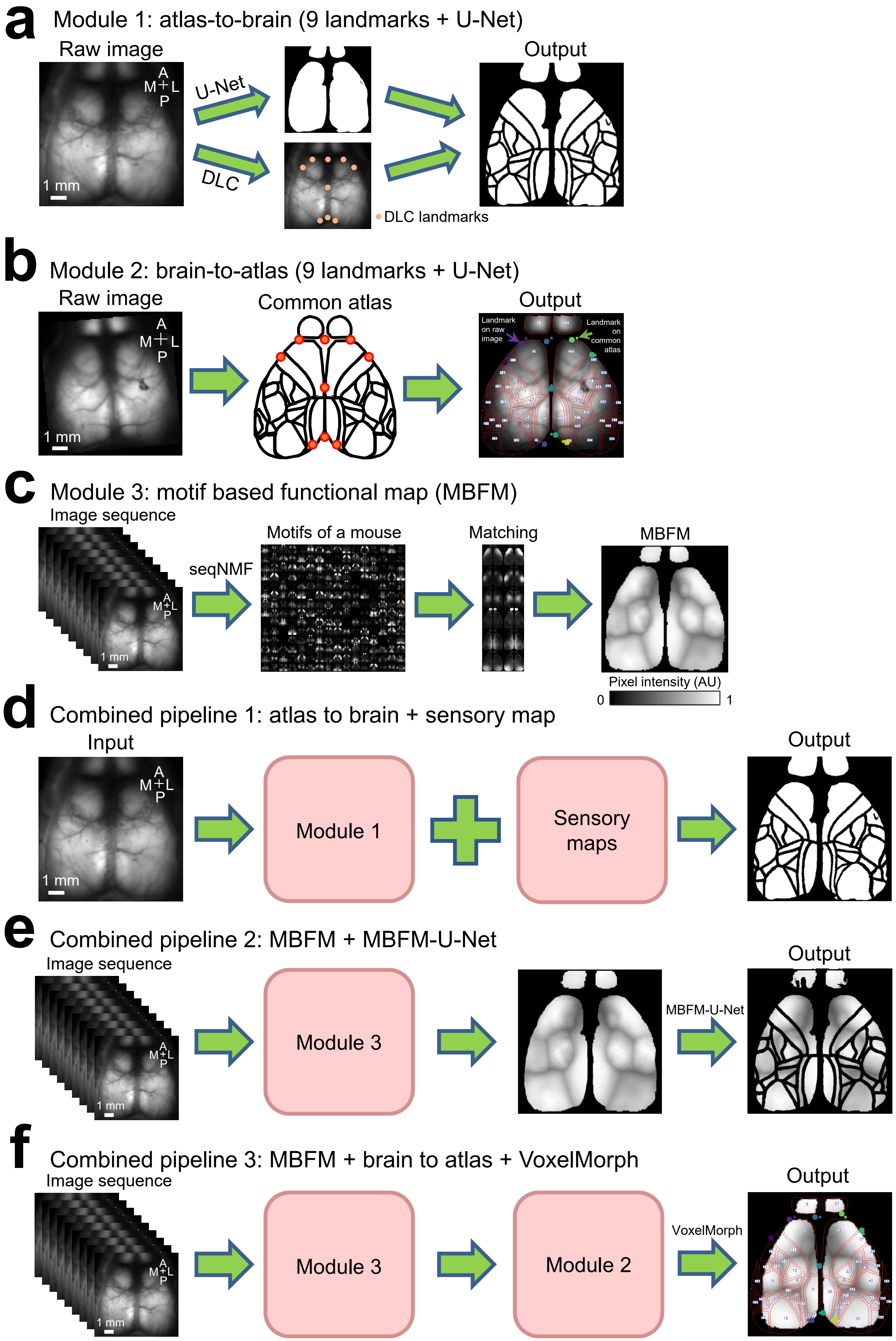
a
Module 1: atlas-to-brain (9 landmarks + U-Net)
Raw image
A
M
L
P
1 mm
U-Net
DLC
Output
DLC landmarks
b
Module 2: brain-to-atlas (9 landmarks + U-Net)
Raw image
Common atlas
Output
Landmark on raw image
Landmark on common atlas
c
Module 3: motif based functional map (MBFM)
Image sequence
seqNMF
Motifs of a mouse
Matching
MBFM
Pixel intensity (AU)
0
1
d
Combined pipeline 1: atlas to brain + sensory map
Input
Module 1
Sensory maps
Output
e
Combined pipeline 2: MBFM + MBFM-U-Net
Image sequence
Module 3
MBFM-U-Net
Output
f
Combined pipeline 3: MBFM + brain to atlas + VoxelMorph
Image sequence
Module 3
Module 2
VoxelMorph
Output

Supplementary Fig. 6. Automated end-to-end pipelines. a Atlas-to-brain pipeline. **b** Brain-to-atlas pipeline. **c** MBFM pipeline. **d** Combination of atlas-to-brain and sensory maps pipeline. **e** MBFM-U-Net pipeline. **f** Combination of MBFM, brain-to-atlas, and VoxelMorph pipeline. We provided Code Ocean capsules to demonstrate the operation of all these automated MesoNet pipelines at 10.24433/CO.1919930.v1, and 10.24433/CO.4985659.v1.

Supplementary Table 1. Definition and common coordinates of landmarks.

Landmarks	Definition	Coordinate (mm)
1	Anterolateral tip of the left parietal bone.	(-3.13, 2.19)
2	Left frontal pole.	(-1.83, 3.41)
3	Posterior tip of the left retrosplenial region.	(-0.85, -4.02)
4	Cross point between the median line and the line which connects left and right frontal pole.	(0, 3.41)
5	Bregma.	(0, 0)
6	Anterior tip of the interparietal bone.	(0, -3.49)
7	Anterolateral tip of the right parietal bone.	(3.13, 2.19)
8	Right frontal pole.	(1.83, 3.41)
9	Posterior tip of the right retrosplenial region.	(0.85, -4.02)

Supplementary Table 2. Comparison of distance between model labelled and human labelled landmarks.

	human1 - human2	model - human1	model - human2
landmarks	Mean distance (mm) ± SEM	Mean distance (mm) ± SEM	Mean distance (mm) ± SEM
landmark 1	0.22 ± 0.03	0.19 ± 0.02	0.18 ± 0.03
landmark 2	0.15 ± 0.01	0.14 ± 0.02	0.16 ± 0.02
landmark 3	0.13 ± 0.02	0.16 ± 0.02	0.12 ± 0.02
landmark 4	0.09 ± 0.01	0.10 ± 0.01	0.10 ± 0.01
landmark 5	0.18 ± 0.02	0.17 ± 0.02	0.14 ± 0.01
landmark 6	0.12 ± 0.02	0.12 ± 0.01	0.13 ± 0.01
landmark 7	0.22 ± 0.02	0.21 ± 0.02	0.19 ± 0.01
landmark 8	0.12 ± 0.02	0.15 ± 0.02	0.12 ± 0.02
landmark 9	0.10 ± 0.01	0.11 ± 0.01	0.10 ± 0.01

Supplementary Table 3. Dataset for model training and testing.

Genotypes	Training set (number of mice)	Test set (number of mice)
GCaMP3	14	4
GCaMP6f	39	4
GCaMP6s	210	14
GFP	4	4
Green reflectance	135	4
PHP.B	0	4
Thy1-GCaMP6	0	4
iGluSnFr	0	4
jrGECO	0	4

Supplementary Table 4. Summary of data augmentation methods.

Transformation	Range
Rotation	20%
Shift	5% both width and height
Shear	5%
Zoom	±5%
Flip horizontally	50% probability
