# Supplementary material for: MesoNet allows automated scaling and segmentation of mouse mesoscale cortical maps using machine learning
Source: Nat Commun. 2021 Oct 13;12:5992. doi: 10.1038/s41467-021-26255-2 (PMC8514445; doi:10.1038/s41467-021-26255-2)
Supplement: Supplementary file 3 — Description of Additional Supplementary Files [file 41467_2021_26255_MOESM3_ESM.pdf]

Description of Additional Supplementary Files

File Name: Supplementary Movie 1
Description: Deformed atlas overlay on motif-based functional maps (MBFMs), n = 37 mice.

File Name: Supplementary Movie 2
Description: Screen capture of code demo video shows five easy-to-use automated pipelines.

File Name: Supplementary Movie 3
Description: Screen capture of code demo video shows automated atlas-to-brain alignment and segmentation of brain images.

File Name: Supplementary Movie 4
Description: Screen capture of code demo video shows automated brain to atlas transformation of brain images.

File Name: Supplementary Movie 5
Description: Screen capture of code demo video shows sensory map-based atlas alignment.

File Name: Supplementary Movie 6
Description: Screen capture of code demo video shows anatomical atlases are directly predicted using a pre-trained MBFM-U-Net model.

File Name: Supplementary Movie 7
Description: Screen capture of code demo video shows atlas alignment using a pre-trained VoxelMorph model.

File Name: Supplementary Movie 8
Description: Screen capture of code demo video shows online training of landmark estimation.

File Name: Supplementary Movie 9
Description: Screen capture of code demo video shows online training of U-Net.

File Name: Supplementary Movie 10
Description: Screen capture of code demo video shows data augmentation.
