# Supplementary material for: MesoNet allows automated scaling and segmentation of mouse mesoscale cortical maps using machine learning
Source: Nat Commun. 2021 Oct 13;12:5992. doi: 10.1038/s41467-021-26255-2 (PMC8514445; doi:10.1038/s41467-021-26255-2)
Supplement: Supplementary file 14 — Reporting Summary [file 41467_2021_26255_MOESM14_ESM.pdf]

nature portfolio

Corresponding author(s): Tim Murphy

Last updated by author(s): Sep 15, 2021

Reporting Summary

Nature Portfolio wishes to improve the reproducibility of the work that we publish. This form provides structure for consistency and transparency in reporting. For further information on Nature Portfolio policies, see our Editorial Policies and the Editorial Policy Checklist.

Statistics

For all statistical analyses, confirm that the following items are present in the figure legend, table legend, main text, or Methods section.

n/a	Confirmed	
☐	☒	The exact sample size (n) for each experimental group/condition, given as a discrete number and unit of measurement
☐	☒	A statement on whether measurements were taken from distinct samples or whether the same sample was measured repeatedly
☐	☒	The statistical test(s) used AND whether they are one- or two-sided<br>*Only common tests should be described solely by name; describe more complex techniques in the Methods section.*
☒	☐	A description of all covariates tested
☐	☒	A description of any assumptions or corrections, such as tests of normality and adjustment for multiple comparisons
☐	☒	A full description of the statistical parameters including central tendency (e.g. means) or other basic estimates (e.g. regression coefficient) AND variation (e.g. standard deviation) or associated estimates of uncertainty (e.g. confidence intervals)
☐	☒	For null hypothesis testing, the test statistic (e.g. F, t, r) with confidence intervals, effect sizes, degrees of freedom and P value noted<br>*Give P values as exact values whenever suitable.*
☒	☐	For Bayesian analysis, information on the choice of priors and Markov chain Monte Carlo settings
☒	☐	For hierarchical and complex designs, identification of the appropriate level for tests and full reporting of outcomes
☒	☐	Estimates of effect sizes (e.g. Cohen's d, Pearson's r), indicating how they were calculated

Our web collection on statistics for biologists contains articles on many of the points above.

Software and code

Policy information about availability of computer code

Data collection	The 12-bit images were captured at a frame rate of 120 Hz (exposure time of 7 ms) with 8 × 8 on-chip spatial binning using EPIX XCAP V3.8 imaging software.
Data analysis	Data was analyzed using GraphPad Prism 6 and custom-written software in MATLAB R2020a and Python 3.7.9. The Python software package, pre-trained landmark estimation model, U-Net model, user's manual, and sample data for the demonstration of MesoNet are available on the public OSF repository https://osf.io/svztu, which also offers a link to the GitHub repository from which the Python package can be installed. We also provide a Google Colaboratory notebook for a fully functional version of the MesoNet command line interface within this GitHub repository (mesonet_demo_colab.ipynb). We also provide demo Matlab code and data to demonstrate the procedures to generate functional maps from spontaneous cortical activity motifs (see "OSF Storage/4_Data_code" at https://osf.io/svztu). Lastly, we provide a Code Ocean capsule to demonstrate the operation of all automated MesoNet pipelines at 10.24433/CO.1919930.v1, and another capsule to demonstrate the MBFM generation process at 10.24433/CO.4985659.v1.

For manuscripts utilizing custom algorithms or software that are central to the research but not yet described in published literature, software must be made available to editors and reviewers. We strongly encourage code deposition in a community repository (e.g. GitHub). See the Nature Portfolio guidelines for submitting code & software for further information.

Data

Policy information about availability of data

All manuscripts must include a data availability statement. This statement should provide the following information, where applicable:

- Accession codes, unique identifiers, or web links for publicly available datasets
- A description of any restrictions on data availability
- For clinical datasets or third party data, please ensure that the statement adheres to our policy

Raw wide-field calcium imaging data generated in this study have been deposited in the Open Science Framework (OSF) public repository (https://osf.io/34uwj). More imaging data and corresponding annotations or masks used for model training (landmark estimation model, U-Net model, MBFM-U-Net model, and VoxelMorph model), source data for figures, and demo data with code have been deposited in another public OSF repository (https://osf.io/svztu). Example brain images for testing the landmark estimation model, U-Net model, MBFM-U-Net model, and the VoxelMorph model, as well as data for the demo videos, are available on the public OSF repository ("OSF Storage/0_Example_data", https://osf.io/svztu).

Field-specific reporting

Please select the one below that is the best fit for your research. If you are not sure, read the appropriate sections before making your selection.

[x] Life sciences   [ ] Behavioural & social sciences   [ ] Ecological, evolutionary & environmental sciences

For a reference copy of the document with all sections, see nature.com/documents/nr-reporting-summary-flat.pdf

Life sciences study design

All studies must disclose on these points even when the disclosure is negative.

Sample size	Sample sizes are determined empirically, and similar in size to most existing studies in the same field. The sample size for the comparison of distance between coordinates of model labelled and manual labelled landmarks was 20 brain images from 20 GCaMP mice. The sample size for the comparison of model-predicted and Otsu's threshold brain delimitation results was 20 brain images from 20 GCaMP mice. The sample size for the comparison of brain-to-atlas alignment was 36 brain images from 36 mice, GCaMP6f, n=4 mice, GCaMP6s, n=4 mice, GCaMP3, n=4 mice, PHP.B, n=4 mice, GFP33,34, n=4 mice, Thy1-GCaMP35, n=4 mice, iGluSnFr 36, n=4 mice, jrGECO37, n=4 mice, Green reflectance on wild type mice, n=4 mice. The sample size for clustering cortical activity motifs was 1194 motifs from 6 mice. The sample size for the comparison of functional alignment pipelines was 14 mice. All of this information is presented in the text. No statistical method was used to predetermine sample size.
Data exclusions	No data were excluded from the analysis.
Replication	For the comparison of distance between coordinates of model labelled and manual labelled landmarks, experiments were repeated 20 times. For the comparison of model-predicted and Otsu's threshold brain delimitation, experiments were repeated 20 times. For the comparison of brain-to-atlas alignment, experiments were repeated 36 times. For the comparison of functional alignment pipelines, experiments were repeated 14 times. All attempts at replication were successful.
Randomization	Training and testing dataset were assigned randomly to the comparison.
Blinding	This was a methodological study using automated procedures and all data were included, eliminating the role of the experimenter in screening data. Animals were not allocated into different groups during data collection and analysis in this study.

Reporting for specific materials, systems and methods

We require information from authors about some types of materials, experimental systems and methods used in many studies. Here, indicate whether each material, system or method listed is relevant to your study. If you are not sure if a list item applies to your research, read the appropriate section before selecting a response.

Materials & experimental systems

n/a	Involved in the study	
x		Antibodies
x		Eukaryotic cell lines
x		Palaeontology and archaeology
	x	Animals and other organisms
x		Human research participants
x		Clinical data
x		Dual use research of concern

Methods

n/a	Involved in the study	
x		ChIP-seq
x		Flow cytometry
x		MRI-based neuroimaging

Animals and other organisms

Policy information about studies involving animals; ARRIVE guidelines recommended for reporting animal research

Laboratory animals	Transgenic GCaMP6f, GCaMP6s, GCaMP3, GFP, Thy1, jrGECO, iGluSnFR, or wild type male mice aged 2–4 months were used in the study.
Wild animals	The study did not involve wild animals.
Field-collected samples	The study did not involve samples collected from the field.
Ethics oversight	Animal protocols (A18-0036 and A18-0321) were approved by the University of British Columbia Animal Care Committee and conformed to the Canadian Council on Animal Care and Use guidelines.

Note that full information on the approval of the study protocol must also be provided in the manuscript.
